# Draft Genome Sequence of the Putative Marine Pathogen Aquimarina sp. Strain I32.4

**DOI:** 10.1128/genomeA.00313-18

**Published:** 2018-04-26

**Authors:** Hilary J. Ranson, Jason LaPorte, Edward Spinard, Andrei Y. Chistoserdov, Marta Gomez-Chiarri, David R. Nelson, David C. Rowley

**Affiliations:** a Department of Biomedical and Pharmaceutical Sciences, University of Rhode Island, Kingston, Rhode Island, USA; b Department of Cell and Molecular Biology, University of Rhode Island, Kingston, Rhode Island, USA; c Department of Biology, University of Louisiana at Lafayette, Lafayette, Louisiana, USA; d Department of Fisheries, Animal and Veterinary Sciences, University of Rhode Island, Kingston, Rhode Island, USA

## Abstract

Aquimarina sp. strain I32.4 (formerly Aquimarina sp. ‘homaria’) is a putative pathogen involved in epizootic shell disease in the American lobster (Homarus americanus). We report here the draft genome sequence for Aquimarina sp. strain I32.4 and describe virulence factors that may provide insight into its mechanism of pathogenicity.

## GENOME ANNOUNCEMENT

Epizootic shell disease (ESD) is a prevalent and uncontrolled disease affecting the carapace of the American lobster (Homarus americanus). ESD is characterized by the formation of lesions on the carapace that exhibit bacterial counts 2 to 4 orders of magnitude higher than those in the surrounding area (1). The cause of ESD is still debated within the scientific literature, but recent studies have suggested that the presence of Aquimarina sp. ‘homaria’ results in the exacerbation of lesions (2–4). Aquimarina sp. strain I32.4 is a Gram-negative chitin-degrading bacterium (5). To aid our understanding of its mechanism of pathogenicity, we announce here the draft genome sequence of Aquimarina sp. I32.4 and describe some of its potential virulence factors.

Aquimarina sp. I32.4 was grown in artificial seawater (Instant Ocean) supplemented with yeast extract (1 g/liter) and peptone (5 g/liter) at 25°C on an elliptical shaker (New Brunswick) for 48 h. Genomic DNA was isolated using the Promega Wizard DNA purification kit, and DNA was resuspended in 2 mM Tris-HCl buffer (Bio Basic, Inc.). DNA was quantified using a NanoDrop 1000 spectrophotometer (ND-1000) and checked for quality on a 1% agarose gel stained with ethidium bromide. DNA was sequenced on an Illumina MiSeq sequencer at the Genomics and Sequencing Center at the University of Rhode Island. Reads were trimmed using the CLC Genomic Workbench (version 8.5.1) for quality, ambiguous base pairs, adaptors, duplicates, and size, resulting in 1,329,308 paired-end reads. The draft genome was assembled using the *de novo* assembly algorithm of SPAdes assembler (version 3.1.1). Contigs with a coverage of >22 reads were processed using the CLC Microbial Genome Finishing module with Aquimarina macrocephali JAMB N27 (NCBI assembly accession number GCA_000520995) used as a reference genome. The completed draft genome is composed of 244 contigs, averaging 20,446 bp in size (total genome, 4,988,869 bp), with an average G+C content of 32.4%. The draft genome was annotated using the Rapid Annotations using Subsystem Technology (RAST) server and resulted in 4,469 open reading frames (6). The closest neighbor identified by SEED viewer 2.0 (7) was Gramella forsetii KT0803 (score, 541).

The genome of Aquimarina sp. I32.4 encodes type III and IV secretion systems. It has been reported that Aquimarina spp. can degrade the carapace of H. americanus (1). Accordingly, 16 putative chitinase genes were found, along with 3 hemolysins and 3 metalloproteases. Three iron acquisition systems were annotated, namely, TonB, including the full complement of proteins responsible for the formation of the TonB-ExbB-ExbD complex, and hemin and ferric siderophores, which were identified and could contribute to virulence associated with this strain. The RAST-annotated draft genome was entered into Antibiotics and Secondary Metabolite Analysis Shell (antiSMASH) for secondary metabolite biosynthesis gene cluster analysis (8). Identification of 10 clusters for secondary metabolism, including T1PKS-NRPS biosynthesis, NRPS, and Trans-ATPKS, were categorized by antiSMASH. Trans-ATPKS biosynthesis produces structurally diverse molecules in other bacteria, including the cytotoxic and antitumor compounds pederin and onnamide (9). Other species in this genus have been shown to have algicidal activity against toxic cyanobacterium strains (10).

### Accession number(s).

This whole-genome shotgun project has been deposited at DDBJ/ENA/GenBank under the accession number PGUB00000000. The version described in this paper is version PGUB01000000.

## References

[1] Chistoserdov AY, Smolowitz R, Mirasol F, Hsu A 2005 Culture-dependent characterization of the microbial community associated with epizootic shell disease lesions in American lobster *Homarus americanus*. J Shellfish Res 24:741–747. doi:10.2983/0730-8000(2005)24[741:CCOTMC]2.0.CO;2.

[2] Whitten MMA, Davies CE, Kim A, Tlusty M, Wootton EC, Chistoserdov A, Rowley AF 2014 Cuticles of European and American lobsters harbor diverse bacterial species and differ in disease susceptibility. MicrobiolOpen 3:395–409. doi:10.1002/mbo3.174. PMC4082712 24817518

[3] Chistoserdov A, Quinn RA, Gubbala SL, Smolowitz R 2009 Various forms and stages of shell disease in the American lobster share a common bacterial pathogen in their lesions. J Shellfish Res 28:689.

[4] Quinn RA, Metzler A, Smolowitz R, Tlusty M, Chistoserdov A 2012 Exposures of *Homarus americanus* shell to three bacteria isolated from naturally occurring epizootic shell disease lesions. J Shellfish Res 31:485–493. doi:10.2983/035.031.0208.

[5] Meres NJ 2016 Chapter 17: Surface biofilm interactions in epizootic shell disease of the American lobster (*Homarus americanus*), p 383–421. *In* Dhanasekaran D (ed), Microbial biofilms—importance and applications. InTech, London, United Kingdom. doi:10.5772/61499.

[6] Aziz RK, Bartels D, Best AA, DeJongh M, Disz T, Edwards RA, Formsma K, Gerdes S, Glass EM, Kubal M, Meyer F, Olsen GJ, Olson R, Osterman AL, Overbeek RA, McNeil LK, Paarmann D, Paczian T, Parrello B, Pusch GD, Reich C, Stevens R, Vassieva O, Vonstein V, Wilke A, Zagnitko O 2008 The RAST server: Rapid Annotations using Subsystems Technology. BMC Genomics 9:75. doi:10.1186/1471-2164-9-75. 18261238 PMC2265698

[7] Overbeek R, Begley T, Butler RM, Choudhuri JV, Chuang H-Y, Cohoon M, de Crécy-Lagard V, Diaz N, Disz T, Edwards R, Fonstein M, Frank ED, Gerdes S, Glass EM, Goesmann A, Hanson A, Iwata- Reuyl D, Jensen R, Jamshidi N, Krause L, Kubal M, Larsen N, Linke B, McHardy AC, Meyer F, Neuweger H, Olsen G, Olson R, Osterman A, Portnoy V, Pusch GD, Rodionov DA, Ru¨ckert C, Steiner J, Stevens R, Thiele I, Vassieva O, Ye Y, Zagnitko O, Vonstein V 2005 The subsystems approach to genome annotation and its use in the project to annotate 1000 genomes. Nucleic Acids Res 33:5691–5702. doi:10.1093/nar/gki866. 16214803 PMC1251668

[8] Weber T, Blin K, Duddela S, Krug D, Kim HU, Bruccoleri R, Lee SY, Fischbach MA, Müller R, Wohlleben W, Breitling R, Takano E, Medema MH 2015 antiSMASH 3.0—a comprehensive resource for the genome mining of biosynthetic gene clusters. Nucleic Acids Res 43:W237–W243. doi:10.1093/nar/gkv437. 25948579 PMC4489286

[9] Piel J, Hui D, Wen G, Butzke D, Platzer M, Fusetani N, Matsunaga S 2004 Antitumor polyketide biosynthesis by an uncultivated bacterial symbiont of the marine sponge *Theonella swinhoei*. Proc Natl Acad Sci U S A 101:16222–16227. doi:10.1073/pnas.0405976101. 15520376 PMC528957

[10] Chen WM, Sheu FS, Sheu SY 2011 Novel L-amino acid oxidase with algicidal activity against toxic cyanobacterium *Microcystis aeruginosa* synthesized by bacterium *Aquimarina* sp. Enzyme Microb Technol 49:372–379. doi:10.1016/j.enzmictec.2011.06.016. 22112563

